# Water meter reading recognition method based on character attention mechanism

**DOI:** 10.1371/journal.pone.0332119

**Published:** 2025-09-24

**Authors:** Shiyu Zhang, Yuanwang Wei, Yonggang Li, Caiying Zhou

**Affiliations:** 1 Provincial Key Laboratory of Multimodal Perceiving and Intelligent Systems, Jiaxing University, ZheJiang, JiaXing, China; 2 School of Information Engineering, Jiangxi College of Applied Technology, Jiangxi, Ganzhou, China; 3 Institute of Information Network and Artificial Intelligence, Jiaxing University, ZheJiang, JiaXing, China; 4 Key Laboratory of Medical Electronics and Health of Zhejiang Province, Jiaxing University, ZheJiang, JiaXing, China; 5 Engineering Research Center of Intelligent Human Health Situation Awareness of Zhejiang Province, Jiaxing University, ZheJiang, JiaXing, China; 6 College of Science, Jiangxi university of Science and Technology, Jiangxi, GanZhou, China; Cairo University, EGYPT

## Abstract

With the rapid advancement of computer vision technology, traditional manual methods of reading meters are increasingly being replaced by automated water meter reading technologies based on image recognition. This technology can precisely locate and recognize the readings on captured images of water meter dials, laying a solid technical foundation for the implementation of remote automatic meter reading systems. However, in practical applications, the recognition of water meter readings still faces challenges due to interference from factors such as shooting angles and changes in environmental lighting. To address these challenges, this paper proposes an innovative method based on deep learning. Firstly, the ResNet-based Feature Pyramid Network (FPN) is used to detect the reading area of the water meter to ensure the accuracy of the detection. For the problem of digit character detection, the character detection attention mechanism is introduced to improve the performance of digit detection and reduce the interference of background noise while ensuring high accuracy. For numerical character recognition, the improved LeNet-5 network can better identify water meter readings in natural scenes. Additionally, the integration of a global average pooling layer within the network effectively alleviates the issue of overfitting. To verify the effectiveness of our method, we conducted experiments on the CCF real-world water meter reading automatic identification dataset. The experimental results show that by scaling the water meter reading area and introducing the character attention mechanism to assist in numerical character detection, the recognition accuracy of individual digits improved by 8.8% and 5.5%, respectively, and the overall recognition accuracy of the final water meter reading also increased by 7.0% and 2.2%. These significant improvements demonstrate the superiority and effectiveness of our method in practical applications.

## Introduction

In recent years, while smart meters are progressively replacing conventional mechanical meters, many regions still retain mechanical meters due to environmental constraints, cost considerations, and other practical factors. Consequently, manual meter-reading remains indispensable, significantly escalating operational costs. With the continuous advancements in water meter metrology and information technology, the detection and recognition of traditional water meter readings have emerged as a critical research focus in the field [1–6]. According to the diversity of detection objectives, existing methods for water meter reading recognition can be broadly categorized into two paradigms: one focuses on detecting and identifying the positional coordinates of dial pointers, and the other is oriented toward the detection and recognition of numerical display regions.

The method based on the detection of the position of the pointer on the water meter dial primarily achieves accurate recognition of the water meter reading by identifying the pointing position of the dial pointer and its corresponding reading [7–10]. In response to the challenges of image damage, Wang et al. [7] proposed a robust pointer reading recognition method. This method enhances the model’s recovery capability against disturbances through designed image corruption augmentation. Additionally, a region-based convolutional neural network (Mask Scoring Region-CNN, MSC R-CNN) was introduced to segment the pointer and the mask on the dial, thereby improving the precision of localization and segmentation. Zhang et al. [8] have put forward an algorithm for recognizing pointer instruments that is applicable to inspection robots for smart substations. By leveraging the localization functions of deep learning, they adopted an instrument classification algorithm based on Faster R-CNN to classify three types of gauges: voltmeters, ammeters, and displays. After the instruments were located, image processing was conducted on the images of the pointer instruments to compute more precise readings. Sun et al. [9] have introduced an automatic reading method for pointer-type instruments based on deep learning. This method initially employs the object detection algorithm YOLOv4 and the Image and Feature Fusion (IFF) module for the localization of the target instrument. Subsequently, it integrates the Character Region Awareness for Text (CRAFT) detection algorithm along with the End-to-End Multi-lingual Text (E2E-MLT) recognition algorithm to identify the scale text and units on the instrument. Wang et al. [10] mitigated the overfitting problems due to insufficient sample quantities and uneven distributions by adopting the K-fold validation method. The approach requires a high degree of accuracy in scale information, consequently setting a higher standard for the treatment of background noise.

The method based on the detection and recognition of the numerical region of a water meter is fundamentally centered around the accurate detection and identification of readings through the recognition of numerical characters within the water meter’s reading area. These methods can generally be categorized into two types. The first type of method tends to directly detect and recognize the entire reading area of the water meter, utilizing temporal connection sequences to accurately determine the position of the numerical characters, thereby completing the identification of the water meter reading [11–14]. However, these methods require a high level of accuracy in the number and positional information of the numerical characters. An error in the detection of the number of digits or a misjudgment in the positional information can both potentially result in erroneous reading recognition. The second type of method, conversely, focuses more on the detection and recognition of individual numerical characters within the reading area of the water meter [15–18]. In response to the challenges associated with half-character recognition, Shen et al. [15] introduced a surrogate character approach that significantly enhances recognition accuracy by modifying the format of the training dataset. This technique leverages adjusted training inputs to better address partial character detection issues, demonstrating a notable improvement in the field of text detection and recognition. Zhang et al. [16] employed an improved line-scan method for accurate recognition of individual segmented numerical characters. Specifically, the method involves scanning from the midpoint of the upper boundary of the digit downward, followed by scanning from the three-quarter and one-quarter midpoints of the left boundary of the digit from left to right. Based on the outcomes of these three scans, the exact digit is determined. This approach requires scanning only three lines, which significantly simplifies the image recognition algorithm, reduces the runtime of the algorithm, and enhances the quality of image recognition. In order to more accurately detect the accuracy of the numerals on the dial of a water meter, Sun et al. [17] proposed an improved Faster-RCNN [19] algorithm for water meter digit detection. This algorithm uses ResNet50 combined with an FPN (Feature Pyramid Network) structure to replace the original ResNet50 as the feature extraction network, which can enhance the model’s accuracy in recognizing small-sized digits. Furthermore, it employs Region of Interest(ROI) Align instead of ROI Pooling to eliminate the errors introduced by the two-step quantization process of ROI Pooling, allowing for more accurate mapping of the candidate regions onto the feature map, thereby further improving the precision of the model. Peng et al. [18] have both adopted an improved deep residual neural network. They trained their models using a dataset that includes both complete character images and half-character images for detection and recognition purposes. However, this approach imposes high demands on the quality of the dataset used for training. Moreover, the method has stringent requirements regarding the positioning of ’half-characters’.

In summary, although significant research achievements and progress have been made in the field of water meter reading recognition, there are still a series of challenges faced in practical applications under natural scenes. For example, readings may be interfered with by complex backgrounds, affected by perspective transformations, and issues such as half-character recognition can arise. To address these difficulties, this paper proposes a natural scene water meter reading recognition method based on a character attention mechanism. This method effectively solves the problem of numerical character detection by introducing a character detection attention mechanism. Additionally, by combining data augmentation techniques and a special tagging method, the accuracy of half-character recognition is further enhanced. With these improvements, higher accuracy water meter reading recognition in natural scenes can be achieved.

## 1 Proposed method

The numeric region of a water meter reading consists of a series of numerical characters arranged in a specific order. The core task in detecting and recognizing water meter readings is to precisely detect these numerical characters and accurately identify them. Subsequently, a complete water meter reading is derived by assembling the recognized digits along with their positional information. In this research, to improve the accuracy of detecting the number of digits and their positional information, the problem of recognizing water meter readings is divided into two sub-problems: the detection of numerical character digits in water meter readings and the recognition of numerical character digits in water meter readings.

Addressing the detection issue of numerical character digits in water meter readings, the character detection attention mechanism method can enhance the performance of character detection, ensuring high accuracy while reducing the interference of background noise. For the issue of numerical character recognition,the improved LeNet-5 [20] network can better solve the problem of water meter reading recognition in natural scenes. In addition, the added token categories can better address the challenge of half-character digit recognition. Moreover, the incorporation of a global average pooling layer in the network effectively mitigates the problem of overfitting.

**Fig 1:**
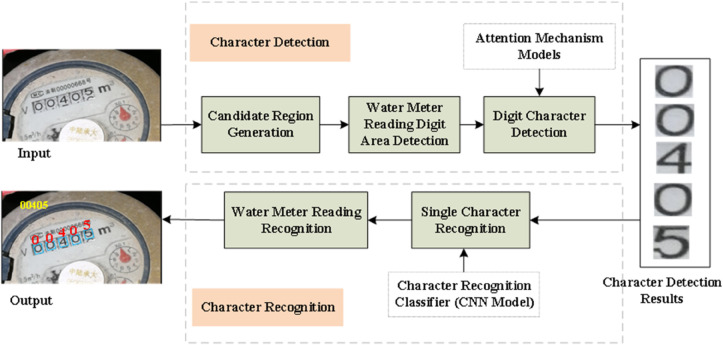
Flowchart of the water meter reading recognition method based on character attention mechanism.

The entire process of our method is depicted in Fig 1. Through this process, the detection and recognition of digital characters in water meter readings can be completed more accurately.

### 1.1 Detection of numerical character digits in water meter readings

#### 1.1.1 Detection of the numerical region in water meter readings.

**Fig 2:**
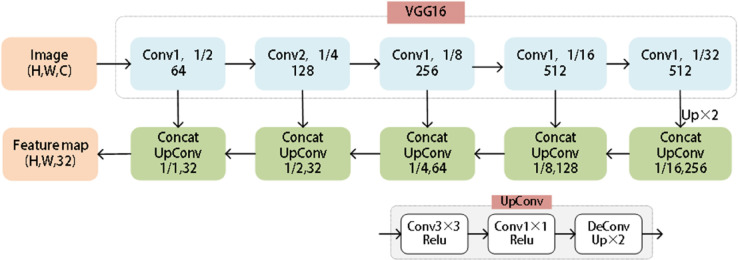
Feature extraction network architecture diagram.

In order to precisely pinpoint the Water Meter Reading Region (WMRR), this study adopts an approach akin to that of DRRG [21], leveraging a shared convolutional network with the VGG16 [22] backbone as the Feature Pyramid Network (FPN) [23] to extract deep features from the WMRR. The architecture of the specific feature extraction network is illustrated in Fig 2.

Direct extraction of the Water Meter Reading Region (WMRR) from the feature map not only captures the numeric information but also includes a significant amount of background noise. To reduce the interference of background noise, this paper introduces a scaling factor, referring to the method in DBNet [21]. By performing a certain proportion of scale transformation on the water meter reading area, a Water Meter Reading Region (WMRDR) with less noise and higher precision can be obtained. The definition of this scaling factor *D* is as follows:

$$D=\frac{S(1-r^{2})}{L}$$ (1)

Within this context, *L* and *S* respectively represent the perimeter and area of the water meter reading region;*r* is the scaling factor, acting as a variable coefficient, which can be adjusted based on different datasets and specific requirements. In this study, it has set *r* to 0.4, aiming to effectively enhance the numeric information and reduce background noise interference by scaling down the water meter reading region.

After obtaining the feature maps through feature extraction and fusion, it uses two convolutional layers to process these features, enabling classification and regression predictions for the water meter reading region and the water meter reading region.

$$CR=conv_{1\times 1}(conv_{3\times 3}(F_{share}))$$ (2)

Among them, $CR\in R^{h\times w\times 8}$. In the above process, four channels are specifically used for the classification and regression tasks of the water meter reading area and the water meter reading area. The other four channels are responsible for the regression calculation of the center point coordinates (*x*,*y*), width (*w*), height (*h*), and rotation angle (*angle*) of the water meter reading region. The central coordinates and the aspect ratio of the length and width of the water meter reading region can be derived from the water meter reading region. Finally, by combining the sample threshold and position aware non maximum suppression (*NMS*) algorithm, the final water meter reading region is predicted. The loss *L* in the detection process mainly consists of two parts: the cross classification loss *L*_*cls*_ and the smooth regression loss *L*_*reg*_:

$$L=L_{cls}+L_{reg}$$ (3)

Among them, *L*_*reg*_ represents the regression loss for the water meter reading region, which is calculated using the smooth *L*1 loss. It includes the width loss *L*_*w*_, height loss *L*_*h*_, and rotation angle loss *L*_*angle*_ of the water meter reading region. The calculation formulas for these are as follows:

$$L_{reg}=L_{h}+L_{w}+L_{angle}$$ (4)

While *L*_*cls*_ is primarily used to enhance the accuracy of the classification of the water meter reading region, the loss in this process is all calculated using OHEM (Online Hard Example Mining). This part is mainly composed of the loss *L*_*r*_, which is specifically designed to calculate the loss of the water meter reading region. To detect a better character region within the water meter reading, an additional loss *L*_*rp*_, which is specifically used to calculate the pixel loss within the water meter reading region, is added on this basis. Additionally, there is the pixel loss *L*_*rn*_ between the water meter reading region and the water meter reading region, which are mainly used to suppress the interference of background noise in these regions. The calculation formula for *L*_*cls*_ is as follows:

$$L_{cls}=L_{r}+\lambda_{1}\times L_{rp}+\lambda_{2}\times L_{rn}$$ (5)

In the formula, $\lambda_{1}$ and $\lambda_{2}$ are weights that vary depending on the dataset, and in the experiments of this paper, these two values are empirically set to 1.0 and 0.5, respectively. After this series of operations, a more accurate water meter reading region and its corresponding region obtained through scaling can be acquired. The subsequent work is primarily based on the water meter reading region.

#### 1.1.2 Correction and detection of numerical characters.

**Fig 3:**
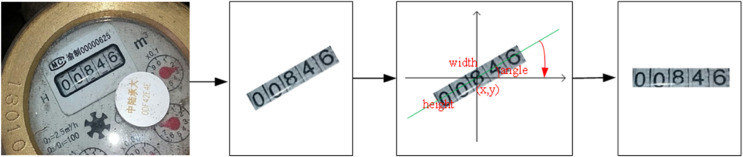
Schematic diagram of horizontal correction for water meter reading region.

After the detection of the water meter reading region, the detected area in some images may be inclined, leading to reduced accuracy in subsequent recognition. To improve recognition rates, we need to apply horizontal correction to the extracted water meter reading region, as illustrated in Fig 3.

Assuming the obtained rectangular information for the water meter reading region is Rect(x,y,width,height,θ), among them, (*x*,*y*) represents the center point of the rectangle, *width* represents the width of the rectangle, *height* represents the height of the rectangle, and the angle formed by the longer axis of the rectangle with the center point (*x*,*y*) and the *x*-axis is *θ*. If the longer axis rotates clockwise, then the angle is negative; otherwise, it is positive. Since the tilt angle *θ* has already been obtained earlier, the reading area of the water meter after the horizontal correction can be obtained by the rotation operation. The rotation matrix can be represented as:

$$M=(\begin{array}{ccc} \cos(\theta ) & -\sin(\theta ) & 0\\ \sin(\theta ) & \cos(\theta ) & 0\\ 0 & 0 & 1 \end{array})$$ (6)

**Fig 4:**
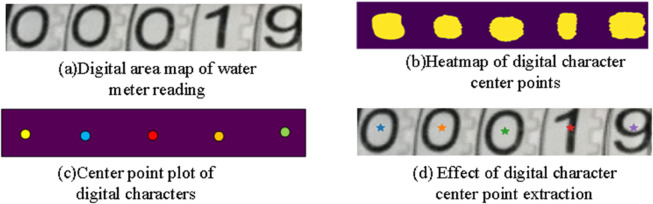
Flowchart for the extraction of numerical character central points.

Typically, the number of numerical characters within a water meter reading region is fixed, and traditional methods often use equal partitioning to detect these characters. However, such methods are susceptible to interference from factors such as the number of numerical characters and shooting distance, which can introduce noise and affect subsequent numerical character recognition. To address this issue, this paper introduces a character attention mechanism during this process, effectively aiding in the extraction of information about the centers of numerical characters. By identifying the centers of numerical characters, The number and spatial distribution of characters within the reading area of the water meter can be detected, thus flexibly adapting to different manufacturers and types of water meter reading scenarios. Additionally, by combining information on the numerical character centers, The digital character area can be located more accurately and effectively reduce the interference of background noise.To obtain information on numerical character centers, a character attention neural network model based on a backbone network was designed, inspired by CRAFT [23]. This model can calculate the areas of interest for numerical characters (i.e., heatmaps of numerical character centers), as shown in Fig 4(*b*). In this heatmap, the value of each pixel represents the probability that the point is a character center. Subsequently, the OUST algorithm (a method of maximum interclass variance) can effectively help to threshold the region of interest for digital characters. After threshold segmentation, The centroid of the region is calculated using the centroid method to identify it as the center of the extracted digital character. Intermediate and result images obtained during the entire process are illustrated in Fig 4(*c*) and Fig 4(*d*).

Next, through the obtained information of the digital area of the water meter reading and the center point of the digital character, the single digital character area within the digital area of the water meter reading can be better detected, as detailed in Algorithm 1. This segmentation method ensures that each segmented numerical character region is determined based on accurate center points, greatly enhancing the precision of the segmentation. Additionally, this approach effectively reduces the interference from background noise, resulting in more accurate detection of numerical characters. The information on the numerical character center points and the numerical character region is crucial for accurately identifying the position of the numerical area, providing a foundation and support for subsequent numerical character recognition and combination.

**Algorithm 1** Numric Character Detection

1: **procedure** NCD (*X*_1_,*Y*_1_,*X*_2_,*Y*_2_,*T*)   ⊳ NCCP$T=\{t_{1},t_{2},t_{3},...,t_{n}\},t_{i}=(x_{i},y_{i})$

2:   For i=1:n do

3:   R←Null

4:   **while** i in WMRR **do**

5:    $H_{1}\leftarrow \text{distance from ti to top edge of WMRR}$

6:    $H_{2}\leftarrow \text{Distance from ti to bottom edge of WMRR}$

7:    $h\leftarrow H_{1}+H_{2}$

8:    $W\leftarrow h_{i}*2/3$

9:    $l_{i}\leftarrow \text{Distiance}(t_{i},t_{i+1})$

10:    $r_{n}\leftarrow (x_{n},y_{n},l_{n},h_{n})$

11:    *r*_*n*_ add to R

12:    return R

13:   **end while**

14: **end procedure**

### 1.2 Recognition of characters in water meter readings

**Fig 5:**
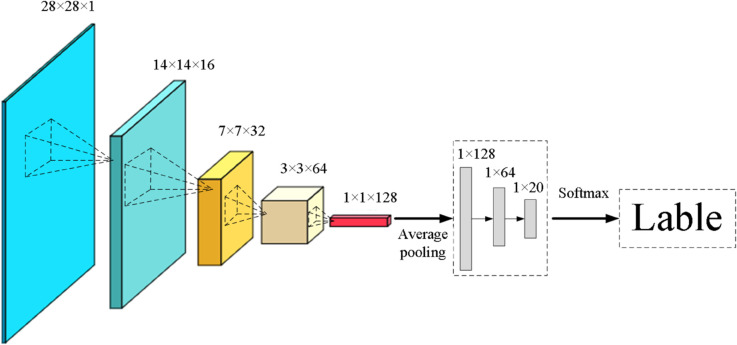
Character recognition network architecture.

Given the limitations in robustness of traditional recognition methods, this paper opts to use convolutional neural networks for the recognition of numerical characters. Considering that the images of the detected numerical character regions are relatively small, using overly complex network structures is impractical. Therefore, this paper references the LeNet-5 network model and has designed an 11-layer convolutional neural network model to accomplish the task of numerical character recognition. The specific architecture of this network is illustrated in Fig 5.

The network model comprises four convolutional layers, four pooling layers, one global average pooling layer, and two fully connected layers, which collectively enable robust feature extraction and classification capabilities.

1) Input Image: The input to this convolutional neural network is a 28x28 pixel image that has been grayscale and normalized.

2) Convolutional Layers (Conv): The network structure designed in this paper includes four convolutional layers. In the first convolutional layer, a 5x5 kernel size (stride=1, pad=2) is used to perform the convolution operation on the input image. For the second, third, and fourth convolutional layers, a kernel size of 3x3 (stride=1, pad=1) is consistently used for convolution calculations. The feature maps outputted by these convolutional layers are used for subsequent recognition tasks.

3) Pooling Layers (Pooling): Following the convolution operations in the convolutional layers, pooling layers are typically introduced to filter and reduce the dimensions of the feature maps, decreasing their size to prevent overly large datasets and enhance computational efficiency. In this network, a 2x2 pooling kernel is used to process each feature map.

4) Global Average Pooling: In the recognition network model of this paper, a global average pooling layer is added after the fourth pooling layer and connected to the subsequent fully connected layers. This step helps to preserve more relevant and important features while discarding less important ones. By doing so, the model better reduces fitting to noise and other irrelevant features, thereby lowering the risk of overfitting and enhancing the model’s generalization ability.

5) Fully Connected Layers (FC): The primary role of this layer is to consolidate the feature maps extracted earlier, integrating and processing them through nonlinear transformations to learn and classify the overall features of the image. This layer typically contains a large number of neurons that receive outputs from the previous layer and produce the final classification results.

6) Output Layer: This layer is responsible for outputting the probability values for different categories. As previously mentioned, the numerical character region is divided into several different categories, so the output layer needs to have several neurons to correspond to these categories. By applying the softmax function, the raw score obtained by the output layer can be converted into a probability distribution and the class with the highest probability is selected as the final classification result. Thus, the values outputted by the output layer are the classification labels for the numerical character regions.

After each digit character is recognized, it is processed by the classification labels and The corresponding coordinate information can accurately assemble the numbers to complete the final identification of the water meter readings. This step ensures the correct combination of digits, avoids recognition errors, and enhances the accuracy and reliability of water meter reading recognition. Finally, the system outputs the combined labels, i.e., the exact reading of the water meter.

## 2 Experiment

### 2.1 Dataset and experimental environment

The dataset used in this experiment is derived from a real-scene water meter reading automatic recognition competition hosted on the DataFountain platform [24]. The dataset includes 1000 training images and 500 testing images, covering both the textual and dial pointer reading areas of water meters. Notably, only the water meter textual reading areas are annotated in the dataset, with annotations including the coordinates of the area and the corresponding recognition results.

During the experiment, we choose Stochastic Gradient Descent (SGD) as the optimizer of the model, and set the initial learning rate to 0.01, and then drop by 0.0001 every 50 epochs. In addition, in order to obtain more data and increase the generalization ability of the model, resizing, flipping, rotating, cropping, and padding Data augmentation operations generate more data to train a more optimized model.. All experiments are carried out on a computer equipped with RTX3080 GPU graphics card and Linux16.04 system, and the experimental environment is Python3.6 and Pytorch1.7.

### 2.2 Label creation

**Fig 6:**
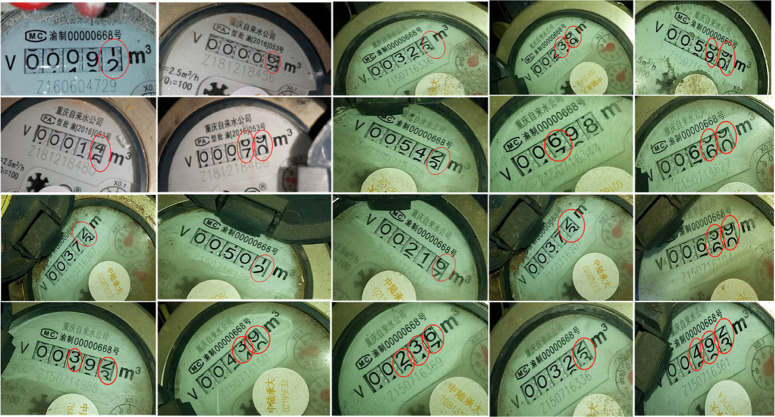
“Half-character” reading example diagram.

Typically, the numerical character regions detected by the segmentation algorithm should contain only one complete digit. However, in practice, it was found that the numerical character regions segmented from water meter readings do not meet this ideal scenario. Instead, most often, multiple “half-character” regions appear, where a single segmented area contains halves of multiple numerical characters. Situations like these “half-character” regions are shown in Fig 6 and pose challenges for subsequent numerical character recognition processing. Therefore, when designing and implementing the numerical character segmentation algorithm, special consideration needs to be given to how to handle these “half-character” regions to improve the accuracy and stability of automatic water meter reading recognition.

In the actual reading process, the recognition results of “half-characters” often cause ambiguities. As shown in the first row and first column of Fig 6, the unit digit in the image is in a state between the numbers 1 and 2. Simply recognizing it as “00091” or “00092” is not accurate enough; the correct reading should be 00091.5. Since errors in the high-position “half-character” readings can lead to significant deviations in the overall reading results, this process cannot be simply regarded as a standard ten-category problem. Through in-depth research on the structure of water meters, it was found that the updates in the water meter reading area are achieved through a rotating axle, connecting the numerical characters in a certain order into a rotation ring. This means that the appearance of “half-character” readings follows certain patterns, divided into ten scenarios in total. To address this challenge, our paper have added ten “half-character” labels on top of the original categories, transforming the overall numerical character area recognition problem into a twenty-category classification issue. It is important to note that “half-character” labels in different positions carry different meanings. For example, the ten-digit label in the second row and second column of Fig 6 initially appears as “19,” but should be correctly recognized as “9” in practice. This requires that throughout the recognition process, it must not only focus on the “half-character” labels themselves but also consider their positions. By combining the position and label information of “half-characters,” the final reading will be more precise. Detailed label categorization is shown in Table 1.

**Table 1:** Table of comparison of character labels.

Character	Label	Character	Label	Character	Label	Character	Label
“0”	0	“5”	5	“0-1”	10	“5-6”	15
“1”	1	“6”	6	“1-2”	11	“6-7”	16
“2”	2	“7”	7	“2-3”	12	“7-8”	17
“3”	3	“8”	8	“3-4”	13	“8-9”	18
“4”	4	“9”	9	“4-5”	14	“9-0”	19

### 2.3 Experimental results and analysis

#### 2.3.1 Experimental results.

To evaluate the overall performance of the proposed method, the aforementioned water meter dataset was used for experimental validation. In the water meter reading area detection stage, three metrics commonly used in text detection are used to measure the detection effect of our method. Namely, precision (P), recall (R), and F-measure(F). In the recognition stage, because the core of the method is the recognition of characters, the recognition accuracy of characters significantly affects the overall recognition results. In order to comprehensively evaluate the performance of the algorithm, not only the overall recognition effect is statistically measured, but also the recognition accuracy of individual characters is analyzed in detail. The specific experimental results are shown in Table 2. The experimental results show that the proposed method achieves 99.8% precision in the detection stage, 97.6% precision in character recognition, and 88.6% precision in text line recognition. In addition, the value of FPS also reflects the excellent performance of our method

**Table 2:** The experimental results of detection and recognition.

	Detection				Recognition		
Index	P	R	F	FPS	Character(P)	Text(P)	FPS
Value	99.80%	98.80%	99.30%	20.02	97.60%	88.60%	20.41

**Fig 7:**
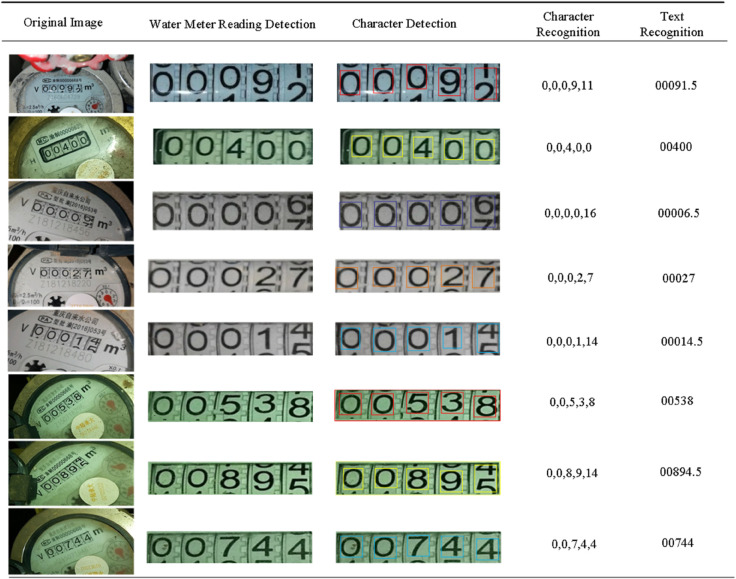
Example diagram of water meter reading recognition results.

In order to better show our experimental results, some experimental results are visualized, including the detection of water meter readings, character detection, character recognition and text recognition. The specific experimental results are shown in Fig 7, which can also find that this method can achieve good results in four stages.

#### 2.3.2 Ablation experiment.

**Fig 8:**
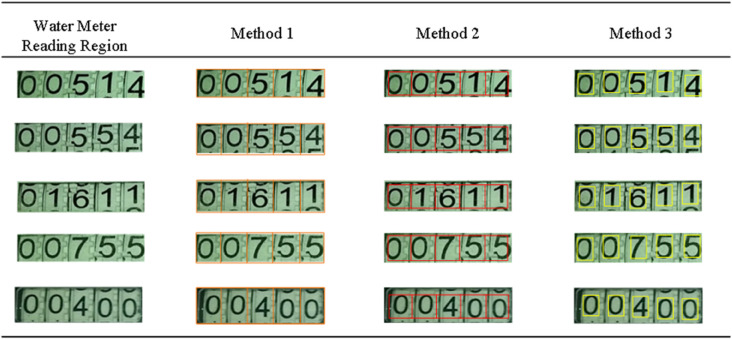
Schematic diagram of detection results from different methods.

As the method proposed in this paper heavily relies on the accurate detection of the water meter reading area, the accuracy of this detection significantly influences the subsequent processes of numerical detection and recognition. To verify the practical effectiveness of the water meter reading area scale transformation and the introduced character detection attention mechanism in the water meter reading detection and recognition task, this paper conducted thorough ablation experiments while controlling other variables. Specifically, Specifically, three digital character detection methods are compared: one based on uniform division of the water meter reading area (Method 1), one incorporating scale transformation of the water meter reading area (Method 2), and one incorporating both scale transformation of the water meter reading area and character detection attention mechanism (Method 3). The experimental results demonstrate that the scale transformation of the water meter reading area and the introduction of the character detection attention mechanism both exhibit significant advantages in numerical character detection. With this method, it can accurately locate the target numerical character area and assist in the segmentation of numerical characters using the numerical character center point information. This not only effectively reduces noise interference but also greatly improves the accuracy of numerical character segmentation, thereby significantly enhancing the accuracy of numerical character recognition. Fig 8 intuitively demonstrates the practical effectiveness of these three numerical character detection methods. A clear comparison shows that compared to Method 1, both Method 2 and Method 3 perform better in terms of detection effectiveness, providing a more solid and reliable foundation for subsequent numerical character recognition. This experimental result fully demonstrates the effectiveness and advancement of the proposed method in the field of water meter reading detection and recognition.

By comparing the example images of the three methods for digit character detection, it can clearly observe their differences in detection effectiveness. Firstly, Method 1 captures the digit regions but also includes a significant amount of background noise during the detection process. These noises will introduce substantial interference in the subsequent digit character recognition stage, leading to reduced precision. Method 2 optimizes the detection of water meter reading regions, notably reducing background noise, especially in the vertical direction. Method 3 effectively suppresses background noise in both horizontal and vertical directions, further enhancing the detection quality. To assess the denoising performance of these three methods more specifically, it conducted ablation experiments and presented the results in Table 3. Through this table, it can more intuitively observe the specific performance and differences of these methods in denoising effectiveness, providing a solid basis for selecting the optimal digit detection method.

**Table 3:** Comparison of recognition precision for characters and text using three different methods.

	Method 1	Method 2	Method 3
Character Recognition(P)	0.888	0.921	0.976
Text Recognition(P)	0.816	0.864	0.886

#### 2.3.3 Analysis and discussion.

From Table 3, it can observe that both the averaging method with water meter reading region scale transformation and the method incorporating character attention mechanism can improve the recognition effectiveness of digit characters to some extent, thereby optimizing the overall water meter reading recognition results. Comparative data also demonstrate that the proposed method effectively aids digit character detection, enhancing the recognition accuracy of digit characters. However, this method also has its limitations. As it is based on digit character detection for recognition, such methods have relatively strict requirements for the detection quantity of digit characters. In practical operations, factors such as insufficient resolution and lighting can easily lead to errors in digit quantity detection, directly resulting in errors in water meter reading recognition, which is an area for improvement in this method.

## 3 Summary

This paper proposes a water meter reading recognition method based on character attention mechanism. By introducing water meter reading region scale transformation and character detection attention mechanism, this method not only maintains high accuracy but also significantly reduces background noise interference. For digit recognition, The improved LeNet-5 network can better meet the needs of water meter reading recognition in natural scenes. Additionally, the incorporation of the global average pooling layer effectively alleviates overfitting issues. To address the challenge of recognizing half-digit characters, The added special label categories can help the recognition better. Experimental results demonstrate that this method has improved accuracy in digit character recognition and water meter reading recognition. However, this method relies on the effectiveness of digit character detection, which is susceptible to environmental factors such as insufficient resolution and lighting changes, potentially leading to errors in digit character quantity detection and subsequently affecting water meter reading accuracy. Therefore, this is an important aspect for future improvement.Future work will mainly focus on two aspects:

1). Addressing the limitations of the current method by seeking solutions to deal with errors in digit character quantity detection caused by factors such as insufficient resolution and lighting changes.

2). Further improving the accuracy of water meter readings, striving to achieve more precise readings to meet higher accuracy requirements in practical applications.
